# Symptom severity and exacerbation frequency in medically treated patients with acromegaly

**DOI:** 10.1007/s11102-026-01732-3

**Published:** 2026-07-23

**Authors:** Eliza B. Geer, David R. Clemmons, Jill Sisco, Maxwell Koobatian, Janetricks C. Okeyo, Tiffany P. Quock, Yang Wang, Raffaella Colzani, Alan Krasner

**Affiliations:** 1 Departments of Medicine and Neurosurgery, Memorial Sloan Kettering Cancer Center, New York, NY USA; 2 Department of Medicine, University of North Carolina at Chapel Hill, Chapel Hill, NC USA; 3 Acromegaly Community, Grove, OK USA; 4 Crinetics Pharmaceuticals, Inc, San Diego, CA USA

**Keywords:** Acromegaly, Patient-reported outcomes, Quality of life, Real-world data, Somatostatin, Symptom exacerbation

## Abstract

**Purpose:**

To quantify the severity, frequency of symptom exacerbations, and associated life impact of acromegaly symptoms in patients treated with injected depot somatostatin receptor ligands (SRLs).

**Methods:**

Patients receiving depot SRLs completed daily 24-hour recall surveys for 3 months. The daily surveys assessed the severity of 7 core symptoms and 2 additional symptoms on numeric scales (0–10). Symptom exacerbation was defined as a ≥ 2-point increase in any symptom score, comparing the current 2-day average with the prior 2-day average. Patients completed a final survey assessing 3-month recall of symptom severity and daily functioning, treatment satisfaction, and healthcare resource utilization (HCRU).

**Results:**

The analysis population included 31 patients: mean (SD) age was 53.4 (13.6) years; 24 (77.4%) were female; 15 (48.4%) were receiving SRL monotherapy and 16 (51.6%) SRL plus non-SRL therapy. Three-month recall overestimated symptom severity relative to daily ratings and did not capture exacerbation frequency. Symptom exacerbations assessed via daily surveys occurred, on average, on 32.1% of days (> 2 days/week) throughout the injection cycle. Greater total and individual symptom severity and exacerbation frequency were related to greater life interference, including impaired daily activities, reduced work productivity, reduced treatment satisfaction, diminished life satisfaction, and increased HCRU.

**Conclusion:**

In patients with acromegaly treated with injected depot SRLs, symptom exacerbations occurred frequently. Symptom severity and exacerbation frequency were associated with impaired daily functioning and reduced life satisfaction. A simple daily symptom assessment provides clinically meaningful information about adequacy of disease control that is not captured by longer-term recall.

**Supplementary Information:**

The online version contains supplementary material available at https://doi.org/10.1007/s11102-026-01732-3.

## Introduction

Acromegaly is a rare disease most commonly caused by a growth hormone (GH)-secreting pituitary adenoma [1, 2]. Excess GH secretion leads to elevated insulin-like growth factor 1 (IGF-I) levels. The combination of elevated GH and IGF-I results in excessive tissue growth and a range of potentially disabling symptoms, such as joint pain, soft tissue swelling, and headache [1]. While approximately 50% of patients achieve long-term remission after pituitary surgery [3, 4], those who do not require indefinite medical therapy to control residual disease activity [2, 5, 6]. Injected long-acting (depot) somatostatin receptor ligands (SRLs; octreotide or lanreotide) are approved first-line medical treatments for persistent disease after surgery [5, 6]. However, monthly depot injections are associated with logistical burdens (e.g., scheduling and traveling for injections), injection site pain, and nodule formation, as well as technical problems with injection technique that may compromise effectiveness [7–10].

Consensus guidelines for medical treatment of acromegaly recommend correction of biochemical abnormalities, stabilizing the residual pituitary tumor, and managing acromegaly symptoms [5, 6]. Although measurements of biochemical control and pituitary tumor size are generally straightforward in clinical practice, limited guidance and tools are available for assessing the adequacy of symptom control. Many patients with medically treated acromegaly report dissatisfaction with the extent of their symptom control, even when biochemical control has been achieved [7–9, 11]. Specifically, patients often describe dissatisfaction with symptom variability or symptoms that intermittently break through or worsen during ongoing medical therapy. Breakthrough symptom exacerbations have been reported retrospectively and appear to follow temporal patterns, (e.g., at the end of the injection cycle or as fluctuating symptom control between injections) [7–9, 11–14].

Assessment of symptom outcomes in acromegaly clinical trials has been inconsistent, using a range of physician and patient-reported outcome (PRO) measures [15–19], which rely on long-term (≥ 1-week) recall of symptom prevalence and/or severity. Symptom severity and day-to-day symptom variability measured with a daily PRO tool have not been previously studied. In addition, the functional life impact of day-to-day symptom variability is unknown. The primary objective of the current study was to quantify symptom severity and variability, based on a daily survey, in patients with acromegaly who were treated with injected depot SRLs. Additional objectives were to evaluate the impact of symptom severity and variability on perceptions of disease burden, daily functioning, treatment satisfaction, and healthcare resource utilization (HCRU).

## Methods

### Study design and patients

Three quantitative, web-based surveys were completed online by medically treated patients aged 18 to 75 years with acromegaly who had undergone transsphenoidal surgery. Patients from the United States, Canada, and the United Kingdom were recruited through patient advocacy groups or social media. Criteria for exclusion from survey participation included current enrollment in a clinical trial or employment by a pharmaceutical manufacturer. Eligibility was confirmed via structured telephone interviews.

The surveys were (1) an initial assessment of patient characteristics (e.g., demographics, acromegaly treatments received, the estimated percentage of time with normal IGF-I), (2) a brief daily symptom survey that was completed for 90 consecutive days, and (3) a final survey of the patient’s experience over the preceding 3-month period. Those patients receiving depot SRL injections who completed the daily symptom survey were included in this analysis.

The daily survey included 9 questions that assessed acromegaly symptoms identified as important to patients in a previous study [20]. The acromegaly symptoms consisted of 7 core symptoms (headache, joint pain, sweating, fatigue, leg weakness, swelling, and numbness/tingling), which were selected based on guidance from the US Food and Drug Administration (FDA), and 2 additional symptoms (difficulty sleeping and difficulty with short-term memory) [20]. Each symptom was rated based on a 24-hour recall period using a scale from 0 to 10, resulting in a total core symptom severity score of 0 to 70, with higher scores indicating greater symptom burden [20].

The final survey was completed based on recall over the last 3 months (during which the daily surveys were completed) and consisted of questions involving disease burden (including symptoms experienced and average severity of those symptoms over the preceding 3 months), treatment experiences, burden of treatment, HCRU, and treatment perceptions. Select results from the final survey were previously published [21].

Institutional review board approval was obtained for the daily symptom survey and final survey questions; all data were anonymized. Patients consented to the analysis of their survey results and received honoraria for their time spent completing the survey.

### Statistical analysis

Symptom variability was expressed as the SD of the total core symptom severity score or as the acromegaly symptom exacerbation frequency (ASEF), defined as the percent of days with at least a 2-point increase in any 1 or more of the 9 acromegaly symptom scores based on comparison of a 2-day average (of day x and day x + 1) to the prior 2-day average (of day x-1 and day x-2). A 2-point difference in an individual symptom score was determined to be meaningful to patients in a separate qualitative research study (data on file).

Summary statistics (e.g., means, SDs) for acromegaly symptom severity scores and ASEFs were calculated. Spearman correlation coefficients and associated nominal *p* values were calculated for associations of symptom severity score or ASEF with patient estimates of the percentage of time their IGF-I levels were normal (from the initial survey) and life impact scores (from the final survey). Relationships between patient-reported treatment satisfaction in alleviation of symptoms, treatment satisfaction in improving quality of life (QoL), and overall treatment satisfaction were also evaluated with Spearman correlation coefficients. Correlation coefficients greater than 0.4 with *p* values < 0.05 for overall scores and < 0.01 for individual symptoms were considered potentially meaningful and statistically significant. There were no adjustments for multiple comparisons.

## Results

### Patients

Of 66 enrolled patients, 31 reported treatment with depot octreotide or lanreotide injections and completed the daily symptom survey, with 30 also completing the final survey. Participants completed 97% of the expected daily data over the 3-month period, and the daily survey completion rate was consistent over time.

Most patients were female (77.4%), White (83.9%), and from the United States (71.0%), and the mean (SD) age was 53.4 (13.6) years (Online Resource 1: Suppl Table 1). The most common self-reported comorbidities were arthritis (45.2%), diabetes (38.7%), and sleep apnea (9.7%). Fifteen patients used SRL monotherapy, and 16 used combination therapy with SRL plus either cabergoline or pegvisomant. During the 90-day survey period, the mean (median; range) number of days between injections (excluding 3 patients who recorded only 1 injection during the survey period) was 27.3 (27.0; 13.8 to 49.0) days. Among the 28 patients who received > 1 SRL injection during the study period, the mean reported dosing interval was ≥ 21 days for 25 patients and 14 to 18 days for the remaining 3 patients. Fifteen (48.4%) patients received > 3 SRL injections during the 90-day survey period (Online Resource 1: Suppl Table 1).

### Symptom prevalence and severity measured using daily survey versus 3-month recall

Based on data from the daily survey, all patients reported at least one acromegaly symptom (based on an item score > 2, the threshold for clinical meaningfulness) during the 90-day survey period. All 9 assessed symptoms were reported at least once by 38.7% of patients. The mean (SD) total core acromegaly symptom severity score (0–70 scale) over the 3-month survey period was 16.4 (11.8), with scores of 17.5 (11.8) for days 1 to 30, 16.1 (12.0) for days 31 to 60, and 15.6 (12.3) for days 61 to 90. The mean severity score for individual symptoms (0–10 scale) ranged from 1.5 to 3.9. There were numerous significant inter-item correlations for symptom severity, including an association between sleep difficulty and short-term memory difficulty (*r* = 0.62, *p* = 0.0002).

**Fig. 1:**
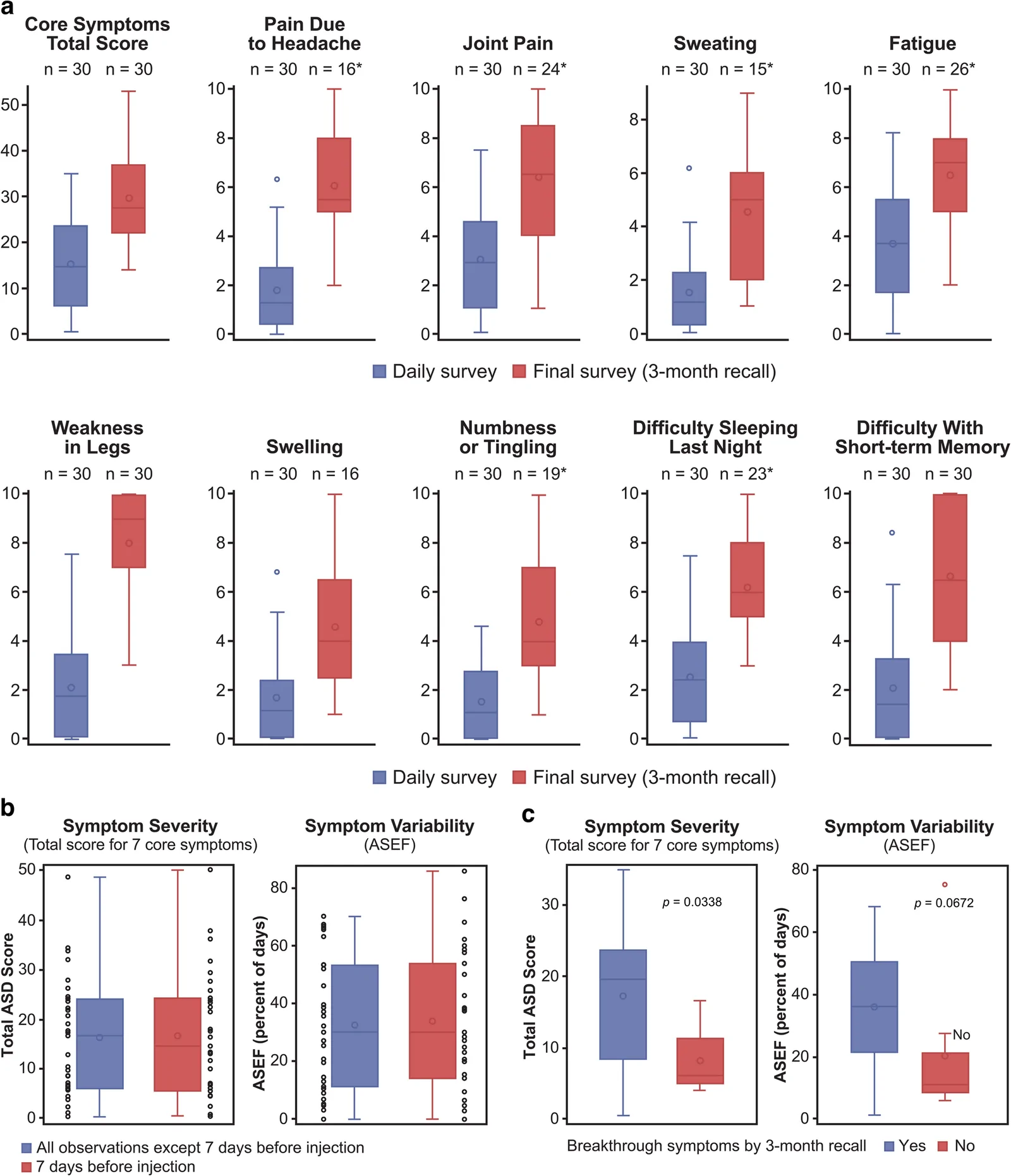
Symptom severity and variability. (**a**) Average severity from the daily survey versus the average from the 3-month recall for the total core symptom severity score and individual item scores, (**b**) Symptom timing in the injection cycle, (**c**) Patients with versus without reported “breakthrough” symptoms at any time prior to the next injection, based on global 3-month recollection in the final survey *This n value reflects the number of patients who endorsed having that symptom on the final survey. A subset of patients who reported an individual symptom on the daily survey failed to report the symptom when responding to the 3-month recall question on the final survey. For the core symptoms total score (but not the individual items), scores for those symptoms that were not endorsed on the final survey were imputed as 0. *P* values are from Wilcoxon rank sum tests of patients with versus without reported breakthrough symptoms. Horizontal lines show median value, boxes show interquartile range, open circles show mean value, and whiskers show minimum and maximum values within the 1.5 IQR above or below Q3 and Q1 values. Any points outside are displayed as outliers. ASD, Acromegaly Symptom Diary; ASEF, acromegaly symptom exacerbation frequency; IQR, interquartile range; Q1, first quartile; Q3, third quartile; SD, standard deviation

For all 9 symptoms assessed, 3-month recall of average symptom severity from the final survey was consistently higher than the average severity reported in the daily symptom surveys (Fig. 1a). In addition, symptoms that were reported in daily surveys with low severity were often omitted from 3-month recall responses. Only 8% to 22% of the individual symptom entries in the daily surveys during the 3-month time period had scores at least as high as the average symptom severity reported by 3-month recall. Average 3-month recall symptom severity values corresponded more closely with maximal severity reported in the daily surveys (Online Resource 2: Suppl Fig. 1).

### Symptom variability

**Fig. 2:**
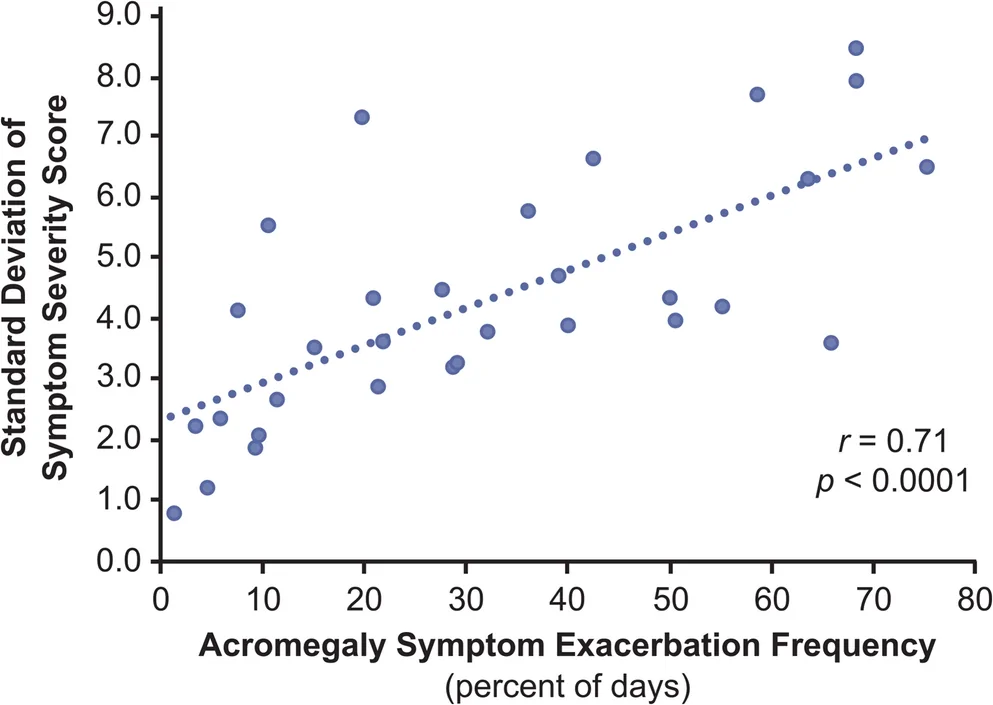
Correlation between the acromegaly symptom exacerbation frequency and the standard deviation of the total core symptom severity score

Symptom variability, as measured by the SD of total core symptom severity scores assessed in the daily survey, was strongly correlated with ASEF (*r* = 0.71, *p* < 0.0001; Fig. 2). Mean total core symptom severity scores from the daily survey correlated with both symptom score SD (*r* = 0.45, *p* = 0.011) and ASEF (*r* = 0.55, *p* = 0.001).

During the survey period, all patients had 1 or more symptoms that worsened, 25 (80.6%) had ≥ 2, and 19 (61.3%) experienced ≥ 3 separate worsening symptoms. A mean of 73.8% of symptom exacerbation days were characterized by 1 symptom worsening, 17.6% by 2 symptoms worsening, and 8.5% by 3 symptoms worsening.

The mean (SD) ASEF for any of the 9 acromegaly symptoms assessed over the 90-day survey period was 32.1% (22.6%) of days, an average of 2.2 days per week. By month, the mean (SD) ASEF was 34.2% (23.1%) for days 1 to 30, 32.1% (23.3%) for days 31 to 60, and 29.6% (26.2%) for days 61 to 90, an average of 2.4 days per week, 2.2 days per week, and 2.1 days per week, respectively. In the subset of patients who received an SRL at injection intervals ≥ 21 days apart (*n* = 25), the mean (SD) ASEF for the 90-day survey period was 34.7% (22.1%). Exacerbations occurred at the greatest frequency for difficulty sleeping (9.1% of days), pain due to headache (7.7%), and fatigue (6.9%; Table 1). Acromegaly symptom exacerbations, which were calculated based on the daily symptom surveys, occurred randomly throughout SRL injection cycles. There was no evidence for an increase in total symptom severity or symptom variability in the week before injection during the assessment period (Fig. 1b).

**Table 1:** Frequency of acromegaly symptom exacerbations

Acromegaly symptom	ASEF Mean (SD) percent of days
Any symptom^a^	
All patients (*n* = 31)	31.6 (4.0)
Injection interval ≥ 21 days apart (*n* = 25)	34.7 (4.4)
Individual symptoms, all patients (*n* = 31)	
Difficulty sleeping	9.1 (1.6)
Headache	7.7 (1.4)
Fatigue	6.9 (1.2)
Sweating	5.3 (1.0)
Joint pain	4.9 (0.9)
Swelling	4.1 (1.0)
Leg weakness	3.9 (0.8)
Numbness/tingling	3.7 (1.0)
Difficulty with short-term memory	3.1 (0.9)

^a^Of the 9 symptoms assessed in the daily survey

*ASEF* acromegaly symptom exacerbation frequency

The exacerbation frequency for sleep difficulty correlated significantly with the severity of all symptoms assessed (*r* values of 0.42 to 0.60; *p* < 0.05), with the exceptions of sweating and difficulty with short-term memory. There were small numerical differences favoring combination therapy but no significant differences in symptom severity or exacerbation frequency when comparing patients on SRL monotherapy to those on combination therapy (Online Resource 3: Suppl Fig. 2).

In the 3-month recall assessed in the final survey, 23 of 31 patients (74.2%) reported at least 1 “breakthrough symptom” (defined as a return of disease symptoms before next dose of medication) occurring in the past 3 months. When asked in the final survey to describe the timing of each breakthrough symptom, 72 of 118 breakthrough symptoms (61.0%) were reported to occur at varying intervals. Other responses included: 1 week before injections (*n* = 16, 13.6% of breakthrough disease symptoms), 2 weeks before injections (*n* = 15, 12.7%), 1 to 2 days before injections (*n* = 6, 5.1%), 3 weeks before injections (*n* = 4, 3.4%), and not sure (*n* = 5, 4.2%).

There were no statistically significant correlations between symptom severity scores by 3-month recall and the overall ASEF from the daily surveys. Patients who reported breakthrough symptoms by the 3-month recall had significantly higher symptom severity scores and nominally higher ASEF, as assessed using daily survey data, compared with those who did not report breakthrough symptoms (Fig. 1c).

### Biochemical control

Biochemical markers were not collected in this online survey study. However, on the initial survey, patients were asked, “On average, approximately what percent of the time do you consider your IGF-1 levels to be in normal range?” The mean (SD) percent of time that patients estimated IGF-I to be normal was 63.2% (34.3%). Self-reported frequency of IGF-I normalization did not correlate significantly with core symptom severity (*r* = -0.30, *p =* 0.102) but did correlate significantly with lower ASEF (*r* = -0.48, *p* = 0.006). Treatment satisfaction with regard to normalization of IGF-I correlated with lower total core symptom severity score (*r* = -0.44, *p* = 0.016).

### Life impact

To evaluate the impact of symptom severity and symptom variability on the patient experience, correlations between total and individual breakthrough symptom metrics with aspects of life interference, daily functioning, work productivity, life satisfaction, treatment satisfaction, and HCRU reported in the final survey were explored (Tables 2 and 3).

**Table 2:** Correlations between symptom severity score and life impact^a^

Survey item/category	Total core symptom severity score
Overall level of health^b^	*r* = -0.51 *p* = 0.004
Interference of acromegaly with life overall^c^	*r* = 0.53 *p* = 0.003
Work productivity affected by acromegaly^d^	*r* = 0.70 *p* = 0.003
Interference of acromegaly with daily functioning^ce^	*r* = 0.53 to 0.70 and *p* < 0.05 for 8 of 15 items
Interference of acromegaly with life satisfaction^ce^	*r* = 0.47 to 0.58 and *p* < 0.05 for 7 of 7 items
Treatment satisfaction^ce^	*r* = 0.44 to 0.57 and *p* < 0.05 for 6 of 10 items

^a^Correlations with *r* value > 0.40 and *p* value < 0.05; see Suppl Table 2 (Online Resource 4) for a complete list of the survey items that were analyzed for an association with symptom severity and ASEF

^b^0 = extremely poor health; 10 = excellent health

^c^0 = no interference; 10 = very significant interference

^d^0 = no effect on work; 10 = completely prevented working

^e^See Suppl Table 2 for a list of survey items in each category

*ASEF* acromegaly symptom exacerbation frequency

**Table 3:** Correlations between acromegaly symptom exacerbation frequency (ASEF) and life impact^a^

Individual item ASEF	Survey item	*r* value	*p* value
Leg weakness	Interference of acromegaly with life overall	0.57	0.001
	Treatment satisfaction for alleviating symptoms of acromegaly	-0.47	0.008
Swelling	Taking care of my children	0.72	< 0.001
	Driving a car	0.54	0.006
Sleep difficulty	Gardening or doing other yard work	0.66	0.004
	Enjoying time with family	0.66	< 0.001
	Enjoying time with friends	0.52	0.004
	Being able to enjoy the moment	0.48	0.008
	Being at peace with yourself	0.52	0.004
	Ability to work outside the home	0.55	0.007
	Treatment satisfaction for alleviating symptoms of acromegaly	-0.47	0.009
	Number of urgent doctor visits related to acromegaly	0.52	0.006
Short-term memory difficulty	Taking care of my spouse/partner	0.72	< 0.001
	Taking care of other members of my family/friends	0.65	0.004
	Enjoying time with family	0.56	0.003
	Number of ER visits related to acromegaly	0.48	0.007

^a^Correlations with *r* value > 0.40 and *p* value < 0.01; see Suppl Table 2 (Online Resource 4) for a complete list of the survey items that were analyzed for an association with symptom severity and ASEF

*ASEF* acromegaly symptom exacerbation frequency, *ER* emergency room

#### Symptom severity

The total core symptom severity score was inversely correlated with self-reported overall state of health (*r* = -0.51, *p* = 0.004) and interference of acromegaly with life overall (*r* = 0.53, *p* = 0.003). Acromegaly symptom severity was also significantly associated with limitations in work productivity (*r* = 0.70, *p* = 0.003) (Table 2) and interference with the following daily activities: ability to work outside the home; taking care of a spouse/partner, children, and other family/friends; driving a car; basic housework; taking family vacations; and overall daily activity (Online Resource 4: Suppl Table 2). In addition, the total symptom severity score correlated with the interference of acromegaly in many aspects of life satisfaction, including being able to enjoy the moment, being at peace with yourself, having a positive feeling, being satisfied with your life, feeling happy, and enjoying time with friends or family (Online Resource 4: Suppl Table 2). Symptom severity was also inversely associated with treatment satisfaction across a number of domains (Table 2).

A formal QoL instrument was not employed in this study. However, higher total core symptom severity was associated with lower acromegaly treatment satisfaction for improving QoL (*r* = -0.49, *p* = 0.006). Treatment satisfaction in alleviation of symptoms was also strongly correlated with treatment satisfaction in improving QoL (*r* = 0.83, *p* < 0.0001). Both treatment satisfaction in alleviation of symptoms (*r* = 0.55, *p* < 0.01) and in improving QoL (*r* = 0.63, *p* = 0.0002) were also strongly related to overall satisfaction with treatment.

The average severity of numerous individual symptoms also correlated with life impact items in each category. With regard to HCRU, the severity of difficulty sleeping correlated with the total number of doctor visits for acromegaly (*r* = 0.48, *p* = 0.007).

#### Acromegaly symptom exacerbation frequency (ASEF)

Overall ASEF (the frequency of any symptom exacerbation measured using the daily survey) correlated with overall life interference (*r* = 0.45, *p* = 0.012) and interference of acromegaly with several life functioning and satisfaction items (the ability to take care of a spouse or partner, children, or other family members/friends; taking family vacations; and enjoying time with family; *r* = 0.47–0.56, *p* < 0.05).

The ASEF of individual symptoms correlated more strongly with life impact items than did the overall ASEF. Of the individual symptoms, the ASEF for sleep difficulty, measured from the daily survey, correlated with the largest number of life impact items (Table 3) and with the number of urgent doctor visits related to acromegaly (*r* = 0.52, *p* = 0.006).

The ASEF for short-term memory difficulty correlated with the frequency of emergency room visits for acromegaly (*r* = 0.48, *p* = 0.007) and the ability to take care of a spouse/partner or other family/friends (Table 3). The ASEF for swelling correlated with difficulty driving a car and taking care of children. The ASEF for weakness in legs correlated with interference with life overall and satisfaction with treatment with respect to alleviation of acromegaly symptoms.

## Discussion

In its guidance on the development of PRO measures, the FDA cautions against relying on memory or asking patients to provide “average” responses reflecting long periods of time in order to avoid undermining content validity (accuracy and comprehensiveness) of the PRO tool [22]. Accordingly, this study assessed symptom severity using a daily (24-hour recall) approach, enabling quantification of day-to-day symptom variability. The Acromegaly Symptom Diary (ASD), designed for daily administration in clinical trials, includes the same acromegaly symptom items used in this survey [20]. Data generated using the ASD [23, 24] were included in the US package insert for paltusotine, an oral selective somatostatin 2 receptor agonist approved for the treatment of adults with acromegaly who had an inadequate response to surgery and/or for whom surgery is not an option [25].

As has been shown previously [7–9], the current study confirms that medical treatment for acromegaly is associated with a persistent symptom burden, which includes frequent periods of acromegaly symptom exacerbations. Further, the magnitude of ongoing symptom severity and exacerbation frequency is associated with interference in daily functioning and reduced life satisfaction.

The use of daily symptom assessments for 90 days was feasible, with a high rate of survey completion (overall, 97% of expected data was collected). Symptom variability was quantified as the ASEF, which reflected a meaningful (i.e., ≥ 2-point) increase across 2-day averages in ≥ 1 acromegaly symptom. Using 2-day averages reduces the influence of random daily changes in symptom severity score (such as the patient’s state when completing the survey) but also minimizes loss of clinically meaningful symptom exacerbations that may be missed in longer-term averages. The ASEF was highly correlated with the symptom scale score SD, a known measure of variability (*r* = 0.71, *p* < 0.0001).

In clinical practice, most routine assessments of acromegaly disease control depend on patient recall of symptom prevalence or severity since the last clinic visit, which may reflect a period of months [8]. Our data show that patient attempts to quantify average symptom severity based on 3-month recall resulted in consistent overestimation relative to severity data collected on a daily basis. Furthermore, less severe symptoms captured on daily surveys were often omitted from responses to 3-month recall questions.

One potential explanation for the quantitative discordance between 3-month recall and daily symptom assessments is that patients disproportionately recall the relatively small proportion of time (8%-22% of days in this study) when they experience their highest symptom severity. It is also possible that symptom exacerbation frequency influences perception of long-term symptom severity. However, the lack of correlation seen in this study between symptom severity assessed by 3-month recall and acromegaly symptom exacerbation frequencies suggests that long-term recall assessments do not adequately describe the full burden of the variable symptom experience in medically treated patients with acromegaly.

Prolonged exposure to elevated levels of IGF-I and GH leads to progressive and serious systemic comorbidities including bone, joint, cardiovascular, metabolic, cerebrovascular, and respiratory disease [1, 26]. For example, soft tissue hypertrophy in the upper airways may cause sleep apnea, cardiomyopathy is a common cardiac effect, and overgrowth of articular cartilage often leads to joint damage. Some symptoms assessed in this study (e.g., sleep difficulty, fatigue, joint pain) may result from acromegaly-induced comorbidities. Mood alterations, particularly depression, are also common in patients with acromegaly [27] and may interact with other symptoms [28, 29].

Among the individual symptoms, the highest exacerbation frequency was observed with sleep difficulty. Variability (ASEF) in sleep difficulty correlated with the severity of most other acromegaly symptoms and with a number of negative life impact items, including decreased ability to work outside the home. Sleep difficulty severity and variability also emerged as the most salient predictor of HCRU (more non-urgent and urgent doctor visits for acromegaly, respectively). The degree to which reported exacerbations in sleep difficulty represent inadequately treated sleep apnea, a complication of soft tissue thickening of the upper airways commonly associated with acromegaly [30], is unknown. The percentage of patients in this analysis with self-reported sleep apnea (9.7%) was lower than expected based on a systematic review of previous studies of acromegaly (23%-25%) [26]. These analyses cannot determine whether there is a causal link between sleep difficulty and the other symptoms or life impact items; however, improvement in sleep quality appears to be an important unmet need in medically treated patients with acromegaly.

Results of this study provide important insights into the nature and pattern of what patients perceive as “breakthrough symptoms.” In previous studies, approximately 50% to 80% of patients with acromegaly receiving injectable SRLs reported breakthrough symptoms occurring toward the end of the dosing cycle [7–9, 31]. In the current study, 67% of patients retrospectively reported breakthrough disease symptoms occurring during the previous 3-month period [21]. However, analysis of daily symptom surveys showed no overall trend for increased symptom severity or variability in the last week of the injection cycle. This finding is consistent with the fact that most patients, when asked directly in the final survey, reported that symptoms occurred at “varied times” in the injection cycle. Average total symptom severity over the entire period of observation was significantly higher among patients reporting breakthrough symptoms in the final survey compared to those who did not report such symptoms. This finding suggests that some patients may perceive “breakthrough” as intermittently severe symptoms occurring despite ongoing treatment, regardless of symptom timing during the injection cycle.

Because this study consisted only of online surveys, IGF-I levels were not collected; therefore, limited information is available on the relationship between biochemical control and symptom control. Reductions in IGF-I and GH levels are generally followed by alleviation of acromegaly signs and symptoms [16–18, 32]. However, some studies have not observed a significant relationship between biochemical control and symptom burden [7–9, 18, 33]. A recent study assessed IGF-I levels and symptoms at 2 timepoints, early and late in the injection cycle, in 20 patients with acromegaly receiving depot SRL injections [12]. Both IGF-I and symptom severity scores were found to be greater in late cycle compared to early cycle, suggesting that disease control wears off as time elapses from the latest depot injection. These findings differ from those of the current study in which symptom exacerbations were observed throughout the injection cycle. One possible explanation for the discordant results is that almost half of patients in the current study received more than 3 injections during the 90-day survey period, indicating that the interval between injections was shorter than 1 month in many patients. It is notable that in the current study, patient self-reported estimates of the percentage of time that IGF-I levels were normal correlated with lower symptom variability but not with symptom severity. Patients’ perception of biochemical control may be influenced by the stability of their symptom experience.

In clinical practice, a second medication (often cabergoline used off-label, or pegvisomant) may be added to an SRL when IGF-I remains above the upper limit of the normal range [5, 34]. In this study, patients using SRL combination therapy had non-significant but numerically lower symptom severity and symptom variability (ASEF) compared with those on SRL monotherapy. It is possible that symptom burden would have been higher in this cohort had these individuals not previously advanced to combination therapy.

Patients with acromegaly have described a profound impact of the disease on their ability to function across several domains of daily living [35]. Both average symptom severity and ASEF were significantly associated with many aspects of life impact, relating to disease interference with daily functioning, life satisfaction, and HCRU (notably for sleep difficulty and short-term memory problems). These findings suggest that approaches to improve patient functioning should consider not only symptom severity but also symptom variability, including exacerbation frequencies.

Interpretation of the findings of this observational study is limited by the inability to compare these results to external published benchmarks, due largely to the unique patient survey tool completed on a daily basis for 3 consecutive months. The high rate of diary completion throughout the study period argues against “diary fatigue.” Furthermore, the average symptom exacerbation frequency was consistently > 2 days per week over the course of the 3-month study observation period. Additional study limitations include lack of formal assessment of health-related QoL and reliance on patient-reported biochemical control information. Formal psychological assessments were not conducted, and the extent to which mood alterations affected the perception of other symptoms is unknown.

Because symptoms are a direct manifestation of a disease state, a simple daily PRO tool may be useful to help patients and physicians systematically measure this important component of disease control simultaneously with biochemical and imaging data collected at routine appointments. Assessing all 3 aspects of disease control may have downstream benefits, including improved functional status, decreased HCRU, and improved health-related QoL. Consistent use of a real-time PRO tool would also establish quantitative targets for reducing symptom severity and exacerbation frequency. Wide adoption of a standard symptom tool for clinical practice may also help address the discordance between PROs and healthcare provider understanding of symptomatic disease control [36].

In conclusion, acromegaly is a chronic disease with a substantial persistent intermittent symptom burden, despite use of depot SRL treatment. Assessment of symptoms on a daily basis in patients with acromegaly receiving a depot SRL demonstrated that symptom exacerbations occurred frequently throughout the injection cycle. Total and individual symptom scores for severity and exacerbation frequency were related to numerous aspects of life interference, including impaired daily activities, reduced work productivity, reduced treatment satisfaction, diminished life satisfaction, and increased HCRU. In patients with acromegaly, a brief daily symptom assessment can provide clinically meaningful information pertaining to adequacy of disease control during medical treatment that complements standard clinical assessments. Further research is warranted to improve methods to measure and achieve symptom control in this disease.

## Supplementary Information

Below is the link to the electronic supplementary material.

Supplementary Material 1

Supplementary Material 2

Supplementary Material 3

Supplementary Material 4

## Data Availability

The datasets used and/or analyzed during the current study are available from the corresponding author upon reasonable request.
