# Supplementary material for: Symptom severity and exacerbation frequency in medically treated patients with acromegaly
Source: Pituitary. 2026 Jul 23;29(4):127. doi: 10.1007/s11102-026-01732-3 (PMC13395975; doi:10.1007/s11102-026-01732-3)
Supplement: Supplementary file 1 — Supplementary Material 1 [file 11102_2026_1732_MOESM1_ESM.pdf]

**Online Resource 1**

**Symptom Severity and Exacerbation Frequency in Medically Treated Patients With Acromegaly**

***Pituitary***

Eliza B. Geer, MD; David R. Clemmons, MD; Jill Sisco; Maxwell Koobatian, PhD; Janetricks C. Okeyo, PhD; Tiffany P. Quock, PhD, MS; Yang Wang, PhD4; Raffaella Colzani, MD; Alan Krasner, MD

Corresponding author:

Alan Krasner, MD

Crinetics Pharmaceuticals, Inc.

akrasner@crinetics.com

**Supplementary Table 1** Patient demographic and clinical characteristics

| | **Patients (*n* = 31)** |
|---|---|
| Age, years, mean (SD) | 53.4 (13.6) |
| Sex, n (%) | |
| Female | 24 (77.4) |
| Male | 6 (19.4) |
| Prefer not to say | 1 (3.2) |
| Race/ethnicity, n (%) | |
| White | 26 (83.9) |
| Hispanic or Latino | 2 (6.5) |
| Asian American | 1 (3.2) |
| Other | 1 (3.2) |
| Prefer not to say | 1 (3.2) |
| Geographic region, n (%) | |
| United States | 22 (71.0) |
| Canada | 6 (19.4) |
| United Kingdom | 3 (9.7) |
| Comorbid conditions (self-reported), n (%) | |
| Arthritis | 14 (45.2) |
| Diabetes | 12 (38.7) |

| | |
|---|---|
| Sleep apnea<br>Carpal tunnel syndrome<br>Heart failure | 3 (9.7)<br>1 (3.2)<br>1 (3.2) |
| Previous pituitary surgery, n (%) | 31 (100) |
| Medical treatment<br>Depot SRL monotherapy<br>Depot SRL + non-SRL monotherapy | <br>15 (48.4)<br>16 (51.6) |
| >3 injections during the 3-month survey period, n (%)* | 15 (48.4) |

*Depot SRLs not approved for administration more frequently than monthly
SRL, somatostatin receptor ligand
