# Supplementary material for: Symptom severity and exacerbation frequency in medically treated patients with acromegaly
Source: Pituitary. 2026 Jul 23;29(4):127. doi: 10.1007/s11102-026-01732-3 (PMC13395975; doi:10.1007/s11102-026-01732-3)
Supplement: Supplementary file 2 — Supplementary Material 2 [file 11102_2026_1732_MOESM2_ESM.pdf]

Online Resource 2

Symptom Severity and Exacerbation Frequency in Medically Treated Patients With Acromegaly

Pituitary
Eliza B. Geer, MD; David R. Clemmons, MD; Jill Sisco; Maxwell Koobatian, PhD4; Janetricks C. Okeyo, PhD; Tiffany P. Quock, PhD, MS; Yang Wang, PhD4; Raffaella Colzani, MD; Alan Krasner, MD

Corresponding author:
Alan Krasner, MD
Crinetics Pharmaceuticals, Inc.
akrasner@crinetics.com

Supplementary Fig. 1 Maximal severity from the daily survey versus the average from the 3-month recall for the total core symptom severity score and for individual item scores

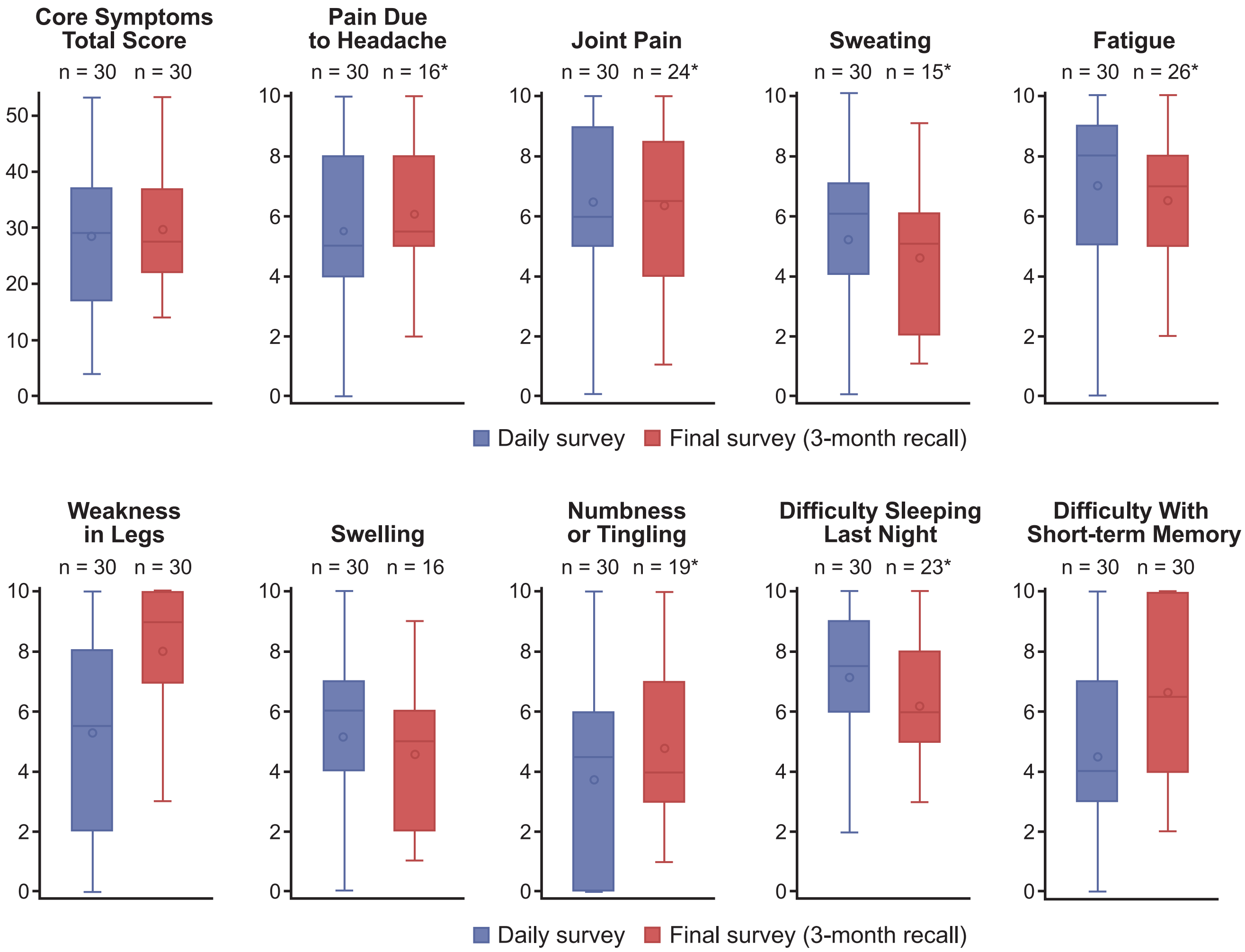

*A subset of patients with the individual symptom on the daily survey failed to report the symptom on the 3-month recall survey. Horizontal lines show median values, boxes show interquartile range, open circles show mean values, and whiskers show minimum and maximum values within the 1.5 IQR above or below Q3 and Q1 values. Any points outside are displayed as outliers. IQR, interquartile range; Q1, first quartile; Q3, third quartile
