# Supplementary material for: Symptom severity and exacerbation frequency in medically treated patients with acromegaly
Source: Pituitary. 2026 Jul 23;29(4):127. doi: 10.1007/s11102-026-01732-3 (PMC13395975; doi:10.1007/s11102-026-01732-3)
Supplement: Supplementary file 3 — Supplementary Material 3 [file 11102_2026_1732_MOESM3_ESM.pdf]

Online Resource 3

Symptom Severity and Exacerbation Frequency in Medically Treated Patients With Acromegaly

Pituitary

Eliza B. Geer, MD; David R. Clemmons, MD; Jill Sisco; Maxwell Koobatian, PhD4; Janetricks C. Okeyo, PhD; Tiffany P. Quock, PhD, MS; Yang Wang, PhD4; Raffaella Colzani, MD; Alan Krasner, MD

Corresponding author:
Alan Krasner, MD
Crinetics Pharmaceuticals, Inc.
akrasner@crinetics.com

Supplementary Fig. 2 Symptom severity and variability during treatment with SRL monotherapy versus combination therapy

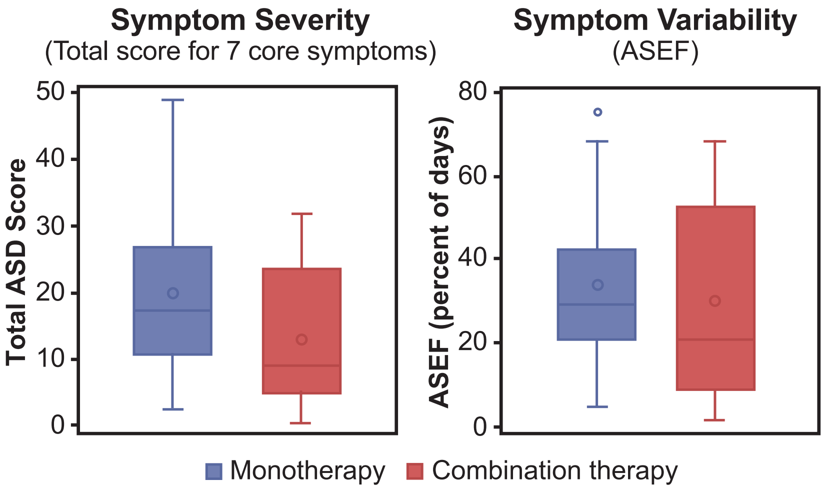

Horizontal lines show median value, boxes show IQR, open circles show mean values, and whiskers show minimum and maximum values within the 1.5 IQR above or below Q3 and Q1 values. Any points outside are displayed as outliers. ASEF, acromegaly symptom exacerbation frequency; IQR, interquartile range; Q1, first quartile; Q3, third quartile; SD, standard deviation; SRL, somatostatin receptor ligand
