# Supplementary material for: Symptom severity and exacerbation frequency in medically treated patients with acromegaly
Source: Pituitary. 2026 Jul 23;29(4):127. doi: 10.1007/s11102-026-01732-3 (PMC13395975; doi:10.1007/s11102-026-01732-3)
Supplement: Supplementary file 4 — Supplementary Material 4 [file 11102_2026_1732_MOESM4_ESM.pdf]

Online Resource 4

Symptom Severity and Exacerbation Frequency in Medically Treated Patients With Acromegaly

Pituitary

Eliza B. Geer, MD; David R. Clemmons, MD; Jill Sisco; Maxwell Koobatian, PhD4; Janetricks C. Okeyo, PhD; Tiffany P. Quock, PhD, MS; Yang Wang, PhD4; Raffaella Colzani, MD; Alan Krasner, MD

Corresponding author:

Alan Krasner, MD

Crinetics Pharmaceuticals, Inc.

akrasner@crinetics.com

Supplementary Table 2 Items from the final survey that were analyzed for an association with symptom severity and ASEF

**Daily functioning**	
Over the past 3 months, how much has your acromegaly interfered with your ability to perform the following activities?<br>(0 = no interference at all; 10 = very significant interference)	Basic housework (e.g., cleaning, laundry, home improvement, etc)
	Taking care of my spouse/partner
	Taking care of my children
	Taking care of other members of my family and friends
	Gardening or doing other yard work
	Driving a car
	Taking family vacations
	Exercising
	Pursuing hobbies you enjoy
	Ability to work outside the home
	Traveling for work
	Ability to go to school (if applicable)
	Intimacy with a partner
During the past 7 days, how much did your acromegaly affect your ability to do your regular daily activities, other than work at a job?	Daily activities

(1 = no effect on my daily activities; 10 = completely prevented me from doing my daily activities)	
**Work productivity**	
During the past 7 days, how much did your acromegaly affect your productivity while you were working?<br>(1 = no effect on my work; 10 = completely prevented me from working)	Work productivity
**Overall health**	
How would you describe your overall level of health?<br>(0 = extremely poor health; 10 = excellent health)	Overall health
**Life overall**	
In the past 3 months, how much would you say that your acromegaly interferes with your life overall?<br>(0 = no interference at all; 10 = very significant interference)	Life overall
**Life satisfaction**	
Over the past 3 months, how much has your acromegaly interfered with your ability to perform the following activities?<br>(0 = no interference at all; 10 = very significant interference)	Enjoying time with family
	Enjoying time with your friends
	Being able to enjoy the moment
	Being at peace with yourself
	A positive feeling
	Feeling happy
	Being satisfied with your life
**Treatment satisfaction**	
How satisfied are you with the following aspects of your acromegaly treatment?<br>(0 = not at all satisfied; 10 = extremely satisfied)	Normalization of IGF-I levels
	Minimizing side effects
	Alleviating symptoms of acromegaly
	Minimizing costs
	Improving quality of life
	Improving ability to perform daily activities
	Mitigating/preventing further complications of acromegaly

	Preventing the need for additional medications
	Reducing the number of medications I have to take
Overall, how satisfied are you with your current prescription acromegaly treatment?<br>(0 = not at all satisfied; 10 = extremely satisfied)	Overall satisfaction with treatment

ASEF, acromegaly symptom exacerbation frequency; IGF-I, insulin-like growth factor 1
